# High-Performance PVC Gel for Adaptive Micro-Lenses with Variable Focal Length

**DOI:** 10.1038/s41598-017-02324-9

**Published:** 2017-05-18

**Authors:** Jin Woo Bae, Eun-Jae Shin, Jaeu Jeong, Dong-Soo Choi, Jong Eun Lee, Byeong Uk Nam, Liwei Lin, Sang-Youn Kim

**Affiliations:** 1 Department of Mechanical Engineering, Berkeley Sensor and Actuator Center, University of California, Berkeley, CA 94720 United States; 2 ATRC, Interdisciplinary Program in Creative Engineering, Korea University of Technology and Education, Cheonan, 31253 South Korea; 3 School of Energy, Materials and Chemical Engineering, Korea University of Technology and Education, Chungnam, 31253 South Korea

## Abstract

This paper presents a bio-inspired adaptive micro-lens with electrically tunable focus made of non-ionic high-molecular-weight polyvinyl chloride (PVC) gel. The optical device mimics the design of the crystalline lens and ciliary muscle of the human eye. It consists of a plano-convex PVC gel micro-lens on Indium Tin Oxide (ITO) glass, confined with an annular electrode operating as an artificial ciliary muscle. Upon electrical activation, the electroactive adhesive force of the PVC gel is exerted on the annular anode electrode, which reduces the sagittal height of the plano-convex PVC gel lens, resulting in focal length variation of the micro-lens. The focal length increases from 3.8 mm to 22.3 mm as the applied field is varied from 200 V/mm to 800 V/mm, comparable to that of the human lens. The device combines excellent optical characteristics with structural simplicity, fast response speed, silent operation, and low power consumption. The results show the PVC gel micro-lens is expected to open up new perspectives on practical tunable optics.

## Introduction

Adaptive focus-tunable micro-lenses driven by various stimuli, such as electrical, thermal, mechanical, and other means, have received considerable attention^1–10^. This is because the adaptive micro-lens can efficiently advance and miniaturize conventional optical systems, which should utilize complicated mechanical parts such as gears, motors, and drivers to allow adjustable focusing and magnification. Recently, emerging liquid-based, variable-focus micro-lenses have been widely investigated in space-constrained applications^4–10^ including cellphone cameras, webcams, endoscopes, and military equipment. The liquid micro-lenses have been demonstrated based on different actuation mechanisms, such as the re-orientation of liquid crystals and electrowetting of liquid droplets. However, these technologies rely on the movement of fluid, which can be subject to external disturbances and fluidic leakages during the operations. Also, solid state-based micro-lenses such as Alvarez lenses are widely reported to have the variable focal length. The Alvarez lenses can achieve focal length tuning by shifting a pair of optical elements, offering large varifocal ranges. However, it still should utilize complicated mechanical parts to realize tunable focal lengths. Therefore, development of smart material and design to open up new perspectives on practical tunable optics is highly desired.

We are interested in an electro-responsive, transparent, and non-ionic polyvinyl chloride (PVC) gel as one of smart polymer gels, which can lead to the considerable achievement of a novel adaptive focus-variable micro-lenses without bulky driving units and the problems associated with using liquids. The PVC gel has three-dimensional crosslinked polymer networks consisting of microcrystallites swollen by the non-ionic plasticizers^11–13^. The end chains of the plasticizers can introduce additional free volume into the PVC polymer chains, which can lead to the disordered arrangement and entanglement of the PVC chains at the junction points, thereby creating numerous microcrystallites in the amorphous region of the physically-crosslinked PVC gel networks without close packing of PVC chains. The PVC physical gel shows an intensive and reversible volume change in response to electrostatic adhesive force (electric-field-induced deformation) from the external electrical stimuli^14–19^. Utilizing the PVC gels responsive to an electric field, we previously reported an innovative approach to realize focus-variable micro-lenses^18, 19^, which are operated by an electric-field-induced deformation of PVC gel toward the anode. In addition, the PVC gel also prevented the evaporation and electrolysis of water during the actuation operation, unlike hydrogels. However, the PVC gel showed poor elasticity or even crushing under a certain pressure due to the lower physical crosslinking network, formed by insufficiently entangled (anchored) polymer chains and few microcrystallites, resulting in deterioration of the focal length variation.

Several factors such as the molecular structure and concentration of the plasticizer in the PVC gel preparation strongly influence the mechanical, dielectric, and electrical properties of the PVC gels^14–19^. In addition, the corresponding results are closely related to the actuation performance of the electroactive PVC gels. To our best knowledge, however, there have been no reports of the molecular weight effect of PVC resin on the electroactive performance of the plasticized PVC gels, which enables higher physical crosslinking density. In this paper, we exploit these ideas and mimic the design of the crystalline lens and ciliary muscle of the human eye to introduce a bio-inspired adaptive PVC gel micro-lens with electrically tunable focus. It consists of a plano-convex PVC gel micro-lens on Indium Tin Oxide (ITO) glass, confined with an annular electrode operating as an artificial ciliary muscle. Upon electrical activation, the electroactive adhesive force of the PVC gel is exerted on the annular anode electrode, which reduces the sagittal height of the plano-convex PVC gel lens, resulting in focal length variation of the micro-lens. The proposed PVC gel showed high transmittance (T ≈ 92.6% at 550 nm), excellent ductility, and electroactive behavior. Based on these properties, our adaptive focus-tunable PVC gel micro-lens had a large relative variation (about 500%) of the focal lengths under an electric field, comparable to that of the human lens. Moreover, the electroactive micro-lens showed fast response speed (≤0.68 sec), silent operation, and low power consumption (12 mW).

## Results and Discussion

### Design and fabrication of the bio-inspired PVC gel micro-lens

**Figure 1:**
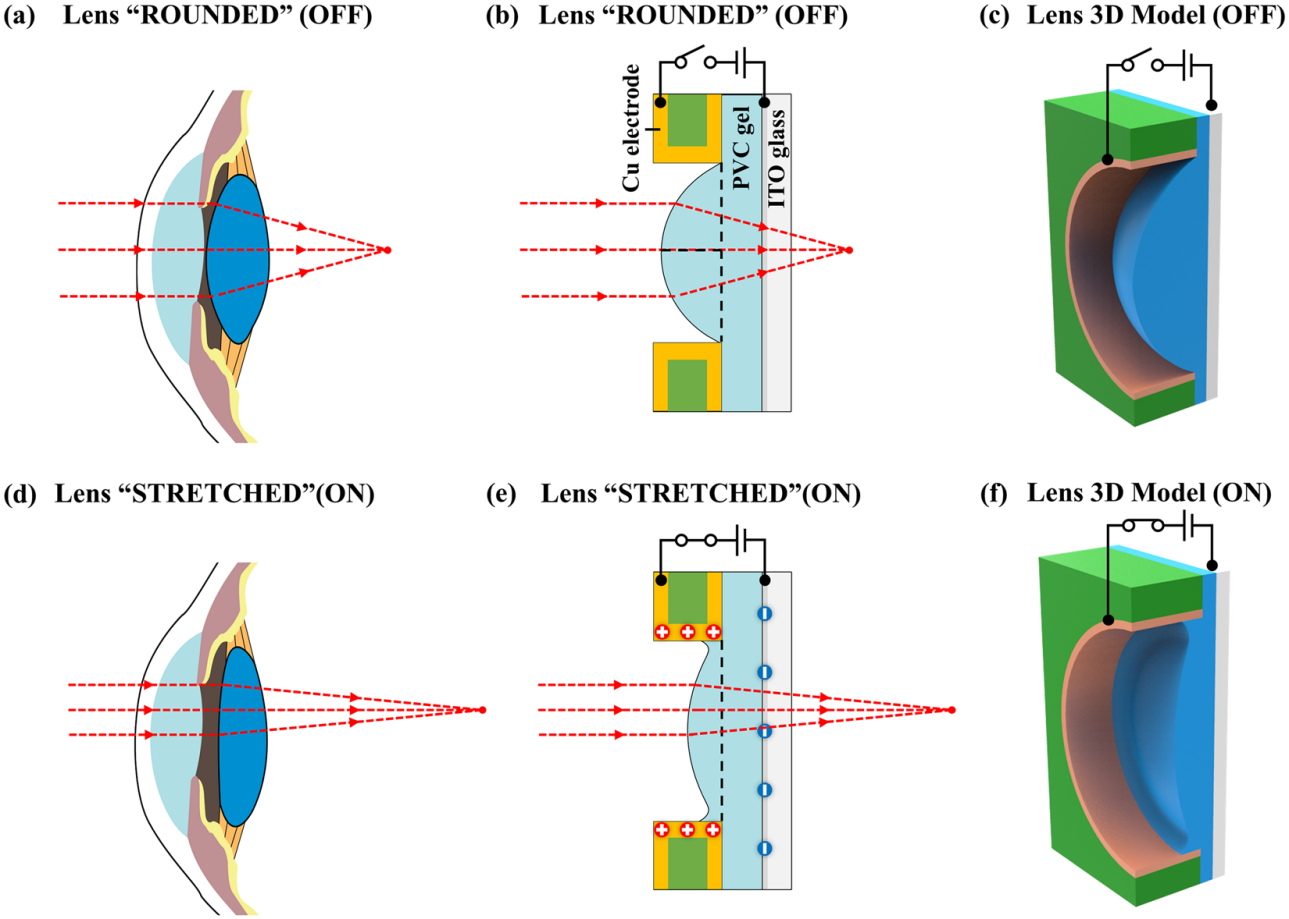
The comparisons between the lens in a human eye and the bio-inspired PVC gel micro-lens. (**a,b**) Schematic sectional views of the two lens systems in an “off” state. (**d,e**) Corresponding views in an “on” state. (**c,f**) Conceptual 3D (three-dimensional) models showing the altered shape of the bio-inspired PVC gel micro-lens under an electric field.

Adaptive PVC gel micro-lens mimics the architecture of the human eye, allowing us to design effective optical devices. Figure 1 shows functional correspondence for the structural arrangement between the human eye lens system and the proposed bioinspired PVC gel micro-lens system. The human lens has an asymmetric biconvex structure^20^, which is stretched or relaxed by the ciliary muscle to adjust the focal length (Fig. 1a,d). A flat PVC gel is mechanically pressed between the ITO glass and a printed circuit board (PCB) with a circular millimeter-scale aperture (Fig. 1b,e) to allow the gel lens to bulge out through the hole to form a plano-convex lens. The 3D (three-dimensional) models (Fig. 1c,f) show the altered shape of the bio-inspired PVC gel micro-lens under an electric field. The optically transparent PVC gel is constructed by a solution-casting process with mixtures of PVC resin and dibutyl adipate (DBA) plasticizer in tetrahydrofuran (THF). Higher mechanical force can results in higher sagittal height of the plano-convex PVC gel lens. As such, the curvature of the PVC micro-lens will respond to the strength of the electric field established by the two electrodes in a similar fashion to the ciliary muscle in a human eye to adjust the focal length.

### Molecular weight effect of PVC polymer on plasticized PVC gels

**Figure 2:**
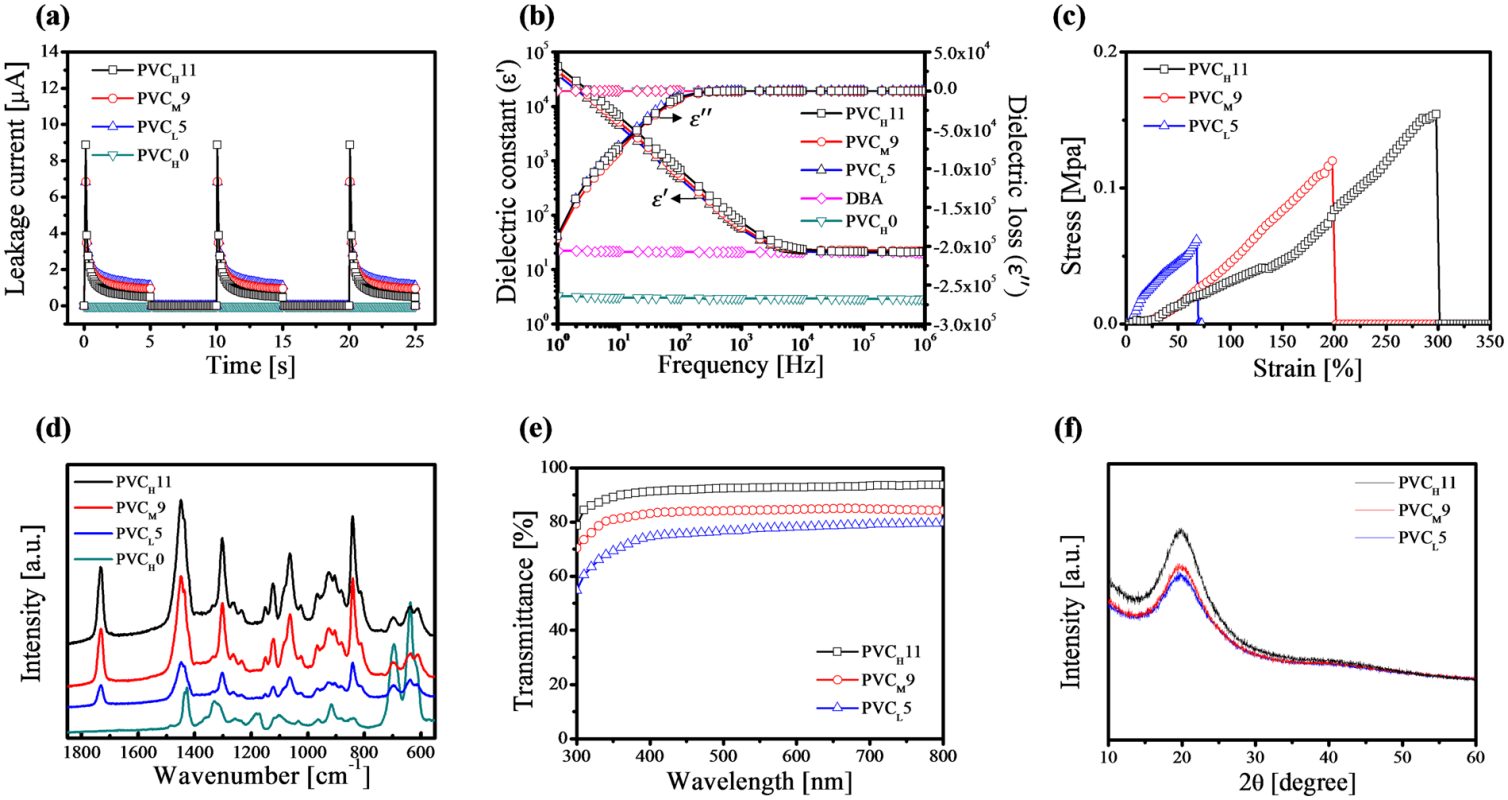
(**a**) Leakage current, (**b**) dielectric constant and loss, (**c**) stress-strain curve, (**d**) Raman spectroscopy, (**e**) UV-Vis spectroscopy, and (**f**) X-ray diffraction patterns of the fabricated PVC gels (PVC_L_5, PVC_M_9, and PVC_H_11).

Various plasticized PVC gels with different molecular weights of PVC polymer and different amounts of DBA plasticizer were investigated (Supplementary Fig. S1) to optimize the mechanical, electrical, and dielectric properties of the plasticized PVC gels. It is found that the leakage current and dielectric loss were both proportionally dependent on the plasticizer concentration but inversely proportional to the molecular weight of the PVC resin. However, the dielectric constant and Young’s modulus increases as the molecular weight of the PVC increases, but decreases as the concentration of the DBA plasticizer increases. By taking into account these effects, we tested different PVC gels denoted as PVC_L_5, PVC_M_9, and PVC_H_11 with optimal plasticizer contents according to the molecular weight of PVC resin and measured structural and optical properties as shown in Fig. 2.

It is found that the pure PVC film is non-conductive under an applied electric field, whereas the PVC gels show measurable currents (Fig. 2a). This is the clear evidence of the direct charge transfer inside the PVC gels under an electric field, resulting in the increases of conductivity in the dielectric material^21^. In addition, PVC gels with higher molecular weight have lower leakage current. It is believed that micro crystallites induced by strongly entangled chains in the high-molecular weight PVC gels could result in the reduction of the leakage current. The conductivity changes in the dielectric material also appear in the results of the dielectric loss in the PVC gels (Fig. 2b). The dielectric constant and loss of the PVC gels increase in the lower frequency region (1–10 kHz) and become nearly constant in the high frequency range (10–1,000 kHz), while the dielectric constant and loss of the DBA plasticizer and the pure PVC film are constants over a wide frequency range. This behavior is believed to be the electrode polarization caused by the increase of conductivity in the dielectric material^21^ as the DBA molecules are charged and polarized in the electric field and the polarized DBA plasticizer facilitates the free dipole re-orientation of the PVC chain molecules in the physically-cross-linked PVC gel networks under a limited frequency range^21^. In spite of the larger amount of DBA plasticizer in the PVC gels, the dielectric loss of PVC gels is almost the same, but the dielectric constant of PVC_H_11 is higher than those of PVC_M_9 and PVC_L_5. As such, the molecular dipolar motion inside the PVC gels is mostly restricted by the molecular weight of the PVC resin instead of the amount of DBA plasticizer. From the simultaneous behavior of dielectric and electric characteristics in the PVC gels, it is concluded that PVC gels with high micro-crystallization induced by the high-molecular-weight PVC allow more free dipole re-orientation of the PVC chain by the DBA plasticizer with lower leakage current, while the non-ionic PVC gel shows the dielectric characteristics for the direct charge transfer.

The PVC_H_11 gel has lower Young’s modulus, higher tensile strength, and extremely high strain at break (almost 300%) as compared to PVC_M_9 and PVC_L_5 gels (Fig. 2c). The superb ductility suggested that the PVC_H_11 gel would be an excellent candidate for electroactive deformation. To further clarify the intermolecular interaction and the internal structure inside the PVC gels, we measured the Raman spectra using a 785 nm laser (Fig. 2d). Although the absorption amount of the C–Cl stretching in the 630 and 688 cm^−1^ region of the PVC gels was similar, aliphatic ketone stretching (C=O) at 1730 cm^−1^ bands gradually grew with the increasing DBA contents in the PVC gels, but their peak position did not shift. The intensity of the spectrum only depended on the concentration of DBA in the PVC gels irrespective of the molecular weight effect of PVC resins. The corresponding results indicate that the fabricated PVC gels were merely mixed with each other as a stable form by physical crosslinking^22, 23^, although the PVC gels had a three-dimensional cross-linked network of flexible PVC chains that contain DBA molecules. In addition, high transparency of the selected PVC gels on the ITO glass was observed across the entire visible spectrum (Fig. 2e), with PVC_H_11 exhibiting the highest transmissivity (T ≈ 92.6% at 550 nm). The transmittance over the entire spectrum is strongly dependent on the DBA plasticizer contents in the PVC gels. Figure 2f shows the XRD patterns of PVC gels, which consist of only a broad halo, indicating an amorphous structure. A broad characteristic diffraction peak is located at 2θ = ~20.0°, which corresponded to an average interlayer spacing of 0.44 nm within micro crystallite from Bragg’s law ($n\lambda =2d\,\sin \,\theta$). The broad peak implies that the micro crystallites were more randomly distributed in the PVC gels without the specific structural modulation^24–26^, which is consistent with its transparent property. It is noteworthy that the peak of amorphous scattering in PVC_H_11 gel is sharper than that in PVC_M_9 and PVC_L_5 gel and the full width at half-maximum (FWHM) of the corresponding diffraction peaks is 5.30° (PVC_H_11), 5.55° (PVC_M_9), and 5.60° (PVC_L_5), respectively. From the Scherrer Equation (${\rm{L}}=k{\rm{\lambda }}/{\rm{\beta }}\,\cos \,{\rm{\theta }}$), it is suggested that the PVC_H_11 gel has a denser (heterogeneous) micro crystallite network structure due to a larger degree of polymer chain aggregation in the amorphous phase^11–13^. However, the thin micro crystallites induced by low-molecular-weight PVC gel were too weak to facilitate the physical cross-linking networks, and thus the PVC gels with low-molecular-weight had low shape stability, poor mechanical strength, and low elasticity. These results suggest that the molecular weight of the PVC resin and the amount of DBA plasticizer strongly affect the gel performances; in particular, PVC_H_11 has the best properties.

### Optical performance of electroactive PVC gel micro-lenses

**Figure 3:**
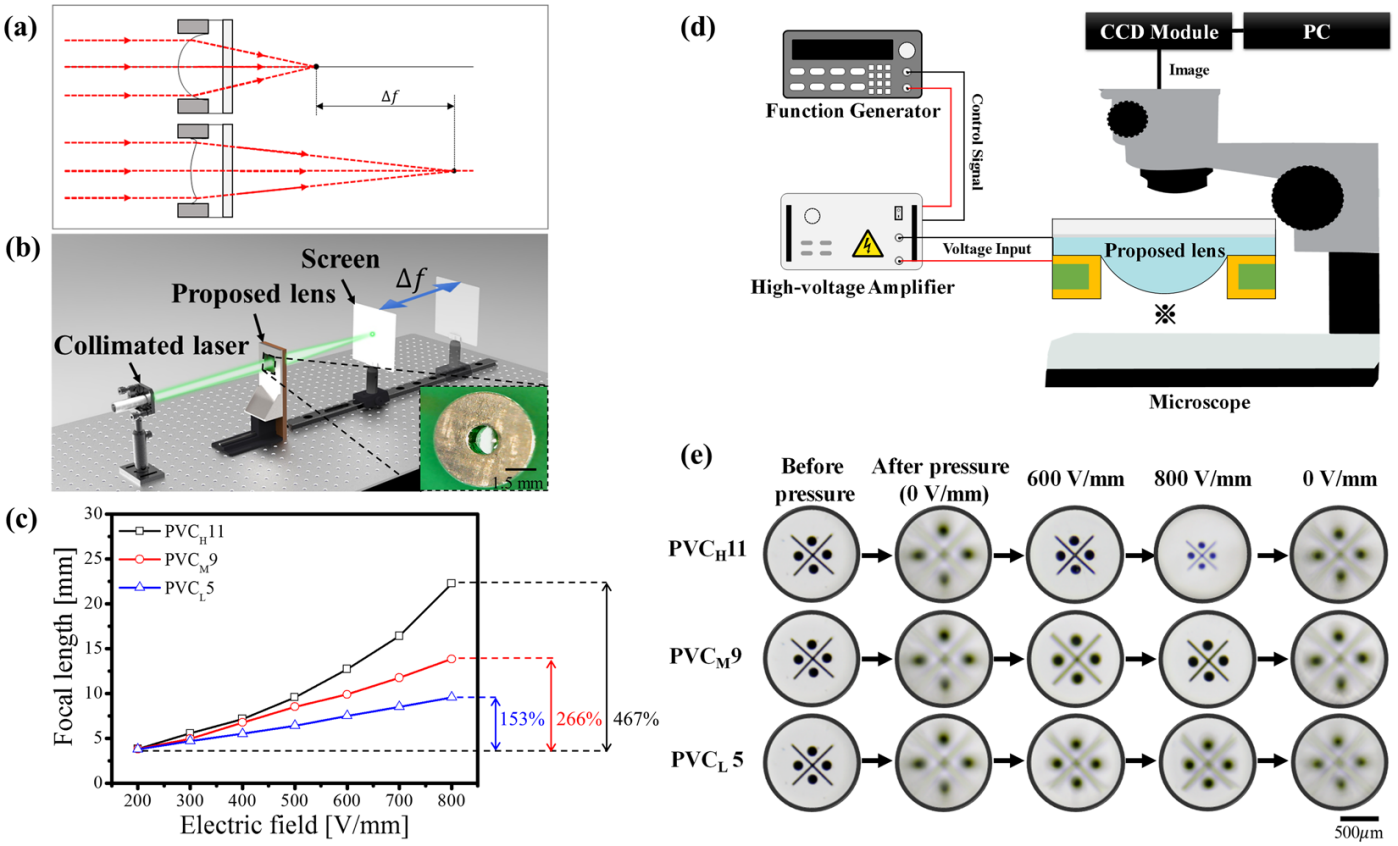
Experimental setup and results of the focal length characterization. (**a**) Illustration of the focal length variation with electric field, (**b**) experimental setup for the focal length measurement and an image (inset) of the fabricated micro-lens, (**c**) measured variable focal lengths versus applied electric field, (**d**) schematic of the electric field-dependent imaging experiment, and (**e**) captured images of a test object; a video clip of this test is available as Supplementary Movie S1.

The refractive indices of the PVC gels were 1.445 (PVC_H_11), 1.447 (PVC_M_9), and 1.453 (PVC_L_5), respectively. From the optical power equation $({\rm{P}}=\frac{1}{{\rm{f}}}\approx ({\rm{n}}-1)(\frac{1}{{{\rm{R}}}_{1}}-\frac{1}{{{\rm{R}}}_{2}}))$, the initial focal lengths (f) of the plano-convex PVC gel micro-lenses were almost the same due to the uniform radius (R) of initial curvature and the similar refractive index (n). The sagittal shape of the plano-convex PVC gel lens is regulated by applying the electric fields and thus the focal length can be tuned as shown in Fig. 3a. Since the refractive index was invariable under an electric field, the variation of the focal length was considered to be merely attributed to the curvature radius of PVC gel deformed by the electric field. The focal length measurement was performed by a custom-built optical layout (Fig. 3b) composed of a collimated light, the proposed PVC gel micro-lens, and a detection screen to confirm the optical performance of the adaptive micro-lens. The proposed micro-lens was placed between the collimated light and the detection screen on linear stages. The collimated light was incident on the convex side of the target lens; the output beam through the other planar side was convergently directed onto the detection screen. The focal length (F) was calculated using equation (1), where A was the distance between the source point of the collimated light and the centre (O) of the plano-convex PVC micro-lens apparatus, and B was the distance between O and the detection screen with the smallest fine spot. As the applied field was changed, the detection screen was moved until the smallest fine spot of collimated light could be observed again, which was indicative of the focal plane. Since the collimated light could be considered to be an infinite beam, in this study, the distance B was considered to be the focal length of the micro-lens^27, 28^.

1 $$\frac{1}{F}=\frac{1}{A}+\frac{1}{B}$$

The initial micro-lens sagitta was 0.5 mm under a suitable external pressure to avoid damage to the gel. When the electric potential was applied between the cathode ITO electrode and the annular anode, the initial micro-lens sagitta was altered by the elect﻿roactive ahdesive force of the PVC gel, which caused the focal length variation of the proposed micro-lenses. The initial focal length of all micro-lenses was fixed at 3.8 mm at 200 V/mm to establish steady plano-convex PVC gels with a uniform radius of initial curvature. Figure 3c shows how the micro-lens adjusts variable focal length under an electric field. The applied electric field was varied from 200 V/mm to 800 V/mm in the increments of 100 V/mm, facilitating continuous increase of the focal length. The focal length of the PVC_H_11, PVC_M_9, and PVC_L_5 micro-lenses increased to 22.3 mm, 13.8 mm, and 9.5 mm, respectively. Compared to the human lens^29^ with a variation of focal length from 43.6 mm to 61.4 mm, the focal length of the bio-inspired the PVC_H_11 was shorter and had a large relative variation (about 500%). The magnitude of current applied to the electric field of 800 V/mm into the PVC_H_11 micro-lens was only 15 μA, which corresponds to the maximum power consumption of only 12 mW. These results demonstrate the suitability of the described approach for a new type of low-power consumption optical devices with variable focal lengths.

The dynamic tuning of the micro-lens at different applied electric fields and the corresponding images are characterized. We used a custom-built optical apparatus (Fig. 3d) composed of a microscope, a PVC gel micro-lens, and a character object “※” attached on the bottom of a PCB. The PVC gel micro-lens was located between the microscope and the character object and the images were acquired by the microscope with a fixed-focus lens. Before applying an appropriate pressure, the initial image through the flat PVC gel at the rest state was focused. During the measurement, the position of the microscope, PVC gel micro-lens, and the character object was fixed. When the pressure-load was applied, and electric fields of 600 V/mm, 800 V/mm, and 0 V/mm were taken, images were captured as shown in Fig. 3e. Because a parabolic curvature of the gel micro-lens was formed, the compressed pressure in the gel caused the image to lose its focus and become blurry. When the electric field reached 600 V/mm, the captured image was in focus for the PVC_H_11 gel micro-lens and appeared less blurry for the PVC_M_9 gel micro-lens. When the applied electric field met 800 V/mm, the image of PVC_M_9 gel micro-lens became acceptably sharp, stable, and focused. In particular, the image captured by the PVC_H_11 gel micro-lens under 800 V/mm gets smaller and blurred than that of 600 V/mm. However, the PVC_L_5 gel became unfocused and significantly blurred in spite of the same electric field. Whenever the electric fields disappeared, the character image of all PVC gel micro-lenses instantly went back to their original scale, out-of-focused and blurry again. The 10,000-actuation cycles of the PVC_H_11 gel micro-lens were also reproducible under the experimental conditions (Supplementary Fig. S2). Therefore, these results demonstrate the varifocal PVC lenses can be applied in optical imaging and the micro-lens of PVC_H_11 gel, rather than that of the PVC_M_9 or PVC_L_5, shows more efficient response to the variation of electric field strength. In addition, neither significant optical aberration, periodical oscillations, significant damage, nor severe surface wrinkling of the actuation membrane was observed during the micro-lens operations (Supplementary Movie S1).

**Figure 4:**
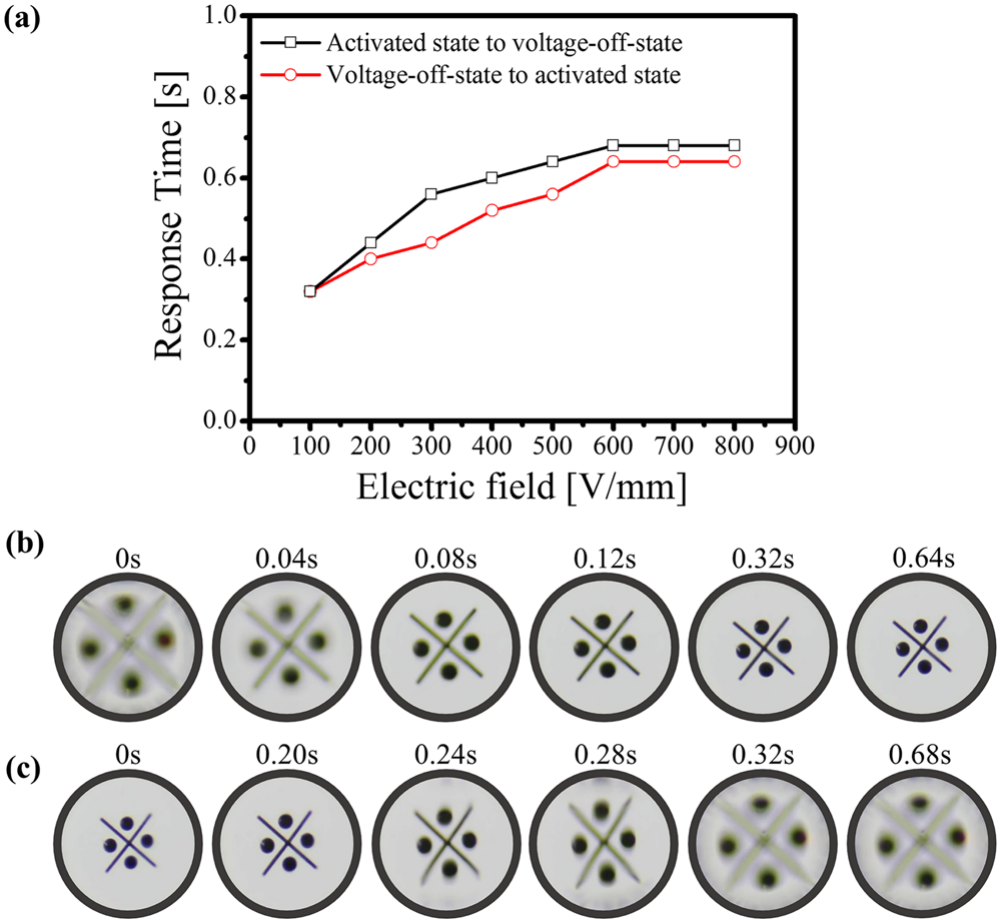
(**a**) Response time as a function of electric field, and the time-lapse images of a character target formed through the adaptive PVC_H_11 gel micro-lenses at (**b**) the on state (600 V/mm) and (**c**) the off state (0 V/mm). The response times at the on state and at the off state for the adaptive PVC_H_11 gel micro-lenses are 0.64 and 0.68 s, respectively.

To demonstrate rapid optical focusing, we conducted dynamic imaging at 25 fps in conjunction with the electrical stimulus. Figure 4 shows the response time as a function of the electric field and time-lapse images of a character target. The response time of the micro-lens in the on or off state of the electrical activation increased with the applied electric field (Fig. 4a). However, the measured response times of the micro-lens at the electric fields above 600 V/mm were almost the same. In particular, the two response times upon turning on (600 V/mm) and off (0 V/mm) of the applied field for the adaptive PVC_H_11 gel micro-lenses were approximately 0.64 and 0.68 s, respectively (Fig. 4b,c) with the backward actuation took slightly longer than the forward actuation. The slight slower time response was due to the lag time required to physically deform the PVC_H_11 gel. However, in comparison with other alternative technologies^30^, the operating speed of the electroactive PVC_H_11 gel micro-lens is relatively fast for applications in consumer electronic devices with completely reversible, silent, and stable operations over repeated measurements.

## Conclusions

The bio-inspired, PVC gel-based micro-lens system is compact, lightweight, electro-responsive, and silent with low power consumptions. In particular, the PVC_H_11 gel used here is optimal in terms of optical, electrical, dielectric, and mechanical properties to facilitate a large electrically tunable range of focal lengths for fast responses. Another attractive feature of the micro-lens is the possible integration with existing electronics and opto-electronics systems because of their compact and simple architecture. Based on the described properties and structural design of the proposed PVC gel micro-lenses, they are potentially suitable to achieve useful optical tuning in various devices as a new generation of electrically tunable optical micro-lenses.

## Methods

### PVC Gel Preparation

Three commercial PVCs were tested, PVC_L_ (Wako), PVC_M_ (Sigma-Aldrich), and PVC_H_ (Scientific Polymer), with molecular weights of 116 K, 230 K and 300 K, and M_W_/M_n_ ratios of 1.82, 2.32, and 2.2, respectively. PVC was first purified by dissolving in tetrahydrofuran (THF, Sigma-Aldrich, 99.9%) and precipitated in methanol. Subsequently, the precipitated PVC powder was filtered and dried. After repeating the procedure three times, prescribed amounts of the purified PVC resin and DBA were dissolved in THF (80% in weight) and thoroughly stirred for 4 h. The PVC gel solution was poured into a Teflon container to allow the evaporation of THF over 1 week, resulting in transparent, flexible, soft, and deformable PVC gels. A wide variety of physically cross-linked PVC gels were made by different weight ratios of PVC resin to DBA plasticizer, 1:0, 1:3, 1:5, 1:7, 1:9, 1:11, and 1:13 and denoted as PVC0 (PVC film), PVC3, PVC5, PVC7, PVC9, PVC11, and PVC13, respectively.

### PVC Gel Characterization

We characterized the mechanical properties of the as-prepared PVC gel using a universal testing machine (UTM, Tinius Olsen, H5KT) according to ASTM D638 type V with a crosshead speed of 50 mm/min at room temperature. The dumbbell-shaped specimens were prepared from the flat drop-casted PVC gel. Impedance spectroscopy measurements were carried out at room temperature to evaluate the polarization effects using an SI-1260 impedance/gain-phase analyzer coupled with a 1296 dielectric interface (Solartron Analytical Co., Farnborough, UK). The samples were measured over the frequency range of 1 Hz to 1 MHz (10 points/decade) with a signal amplitude of 2 V at room temperature. Two parallel copper (Cu) plate electrodes of 10 mm in diameter were used to prepare the circular shape PVC gel specimens of about 10 mm in diameter, and the thickness of the PVC gels was measured by a digital thickness meter. The measurement of leakage current was carried out with a potentiostat/galvanostat (Bio-logic Science Instruments, SP300) under an applied electric fields of 800 V/mm at room temperature. The optical transmittance of the PVC gels onto the ITO glass was measured using an ultraviolet–visible (UV-Vis) spectrophotometer (HP 8452, HP, USA). To investigate the molecular interactions between the PVC and DBA plasticizer, Raman spectroscopy with a Renishaw InVia system (785 nm, 40 mW at the sample) was performed on the PVC gel surface with a 0.75 numerical aperture lens on an inverted microscope. X-ray diffraction (XRD) analysis was carried out with an X-ray diffractometer (Empyrean, PANalytical) at 40 kV and 30 mA to examine the structural changes of the PVC gels. The refractive indices of the PVC gels were evaluated by an Abbe refractometer (ATAGO NAR-4T Solid, Kirkland, WA, USA).

### Micro-lens Fabrication

The as-prepared PVC gel, tailored to uniform 1 mm in thickness, was gently placed on the top of the transparent glass substrate coated with ITO. Afterwards, a 1-mm-thick printed circuit board (PCB), having a Cu-plated through-hole structure of 1.5 mm in diameter was placed on the PVC gel and gently pressed to accomplish the assembly process. This resulted in the conversion of the flat PVC gel to a plano-convex lens with the initial sagitta of 0.5 mm and the chord length of 1.5 mm, which corresponded to the initial radius (0.813 mm) of the PVC gel micro-lenses.

### Micro-lens Performance

The focal length experiments of the PVC gel micro-lens were carried out in a dedicated custom-built optical apparatus, composed of a collimated laser, a plano-convex PVC gel micro-lens, and a detection screen with a graph paper. The focusing performance experiment of the PVC gel micro-lens was conducted in ambient conditions. The electric potential was applied between the ITO electrode and the Cu electrode in the middle of PCB substrates. We first characterized the variations of the apparent focal length of the micro-lens with increasing the electric field. Due to the migration of the PVC gel micro-lens toward the annular anode electrode under electrical activation, the performance of the actual PVC gel micro-lens was measured in the effective diameter of 1.2 mm. The potential was altered from 200–800 V/mm in the increments of 100 V/mm for 5 s in each point, and then switched off. The current was monitored using an oscilloscope (Tektronix, TDS 1012B).

We also examined the optical characteristics of the PVC gel micro-lens using a custom-built optical apparatus composed of a stereo binocular microscope (Sunny, SZM45T-B1), a PVC gel micro-lens apparatus, and a transparent film marked with a ‘※’ character on the bottom of the ITO glass slide. Using the microscope, the character image (※) was first brought into focus with the uncompressed PVC gel before applying an external electric filed. Then after allowing the plano-convex PVC gel micro-lens by compressing the apparatus, a single pulse input of 600 V/mm was applied for 5 s and then another single pulse of 800 V/mm for 5 s. Between the two inputs, there was a rest state (0 V/mm) for 5 s. During this experiment, we observed the resulting images. We also demonstrated the transient responses of the proposed PVC gel micro-lenses at both the activated and the rest states. The response time was confirmed by a camera (with a frame rate of 25 fps) with a step function of the input electric field, whose magnitude is altered in the increments of 100 V/mm up to 800 V/mm with an on-off interval of 60 s. To verify the lifetime of the PVC_H_11 gel micro-lens, the electric field of 800 V/mm was applied to the proposed gel lens during 5 s and then the electric field of 0 V/mm was provided to the lens for 5 s. This procedure was conducted 10,000 times.

## Electronic supplementary material

Supplementary Information

Supplementary Movie S1
