# Supplementary material for: High-Performance PVC Gel for Adaptive Micro-Lenses with Variable Focal Length
Source: Sci Rep. 2017 May 18;7:2068. doi: 10.1038/s41598-017-02324-9 (PMC5437028; doi:10.1038/s41598-017-02324-9)
Supplement: Supplementary file 1 — Supplementary Information [file 41598_2017_2324_MOESM1_ESM.doc]

**Supplementary Information**

**High-Performance PVC Gel Adaptive Micro-Lenses with Variable Focal Length**

**Authors:**

Jin Woo Bae^1,*^, Eun-Jae Shin^2,*^, Jaeu Jeong^2^, Dong-Soo Choi^2^, Jong Eun Lee^3^, Byeong Uk Nam^3^, Liwei Lin^1^, and Sang-Youn Kim^2^

**Affiliations:**

1: Department of Mechanical Engineering, Berkeley Sensor and Actuator Center, University of California, Berkeley, CA 94720, United States;

2: ATRC, Interdisciplinary Program in Creative Engineering, Korea University of Technology and Education, Cheonan 31253, South Korea;

3: School of Energy, Materials and Chemical Engineering, Korea University of Technology and Education, Chungnam 31253, South Korea;

^*^Correspondence should be addressed to S.Y.K. (email: sykim@koreatech.ac.kr)

**SUPPLEMENTARY FIGURES**

**Various properties of plasticized PVC gels with different molecular weights of PVC polymer and amounts of DBA plasticizer**


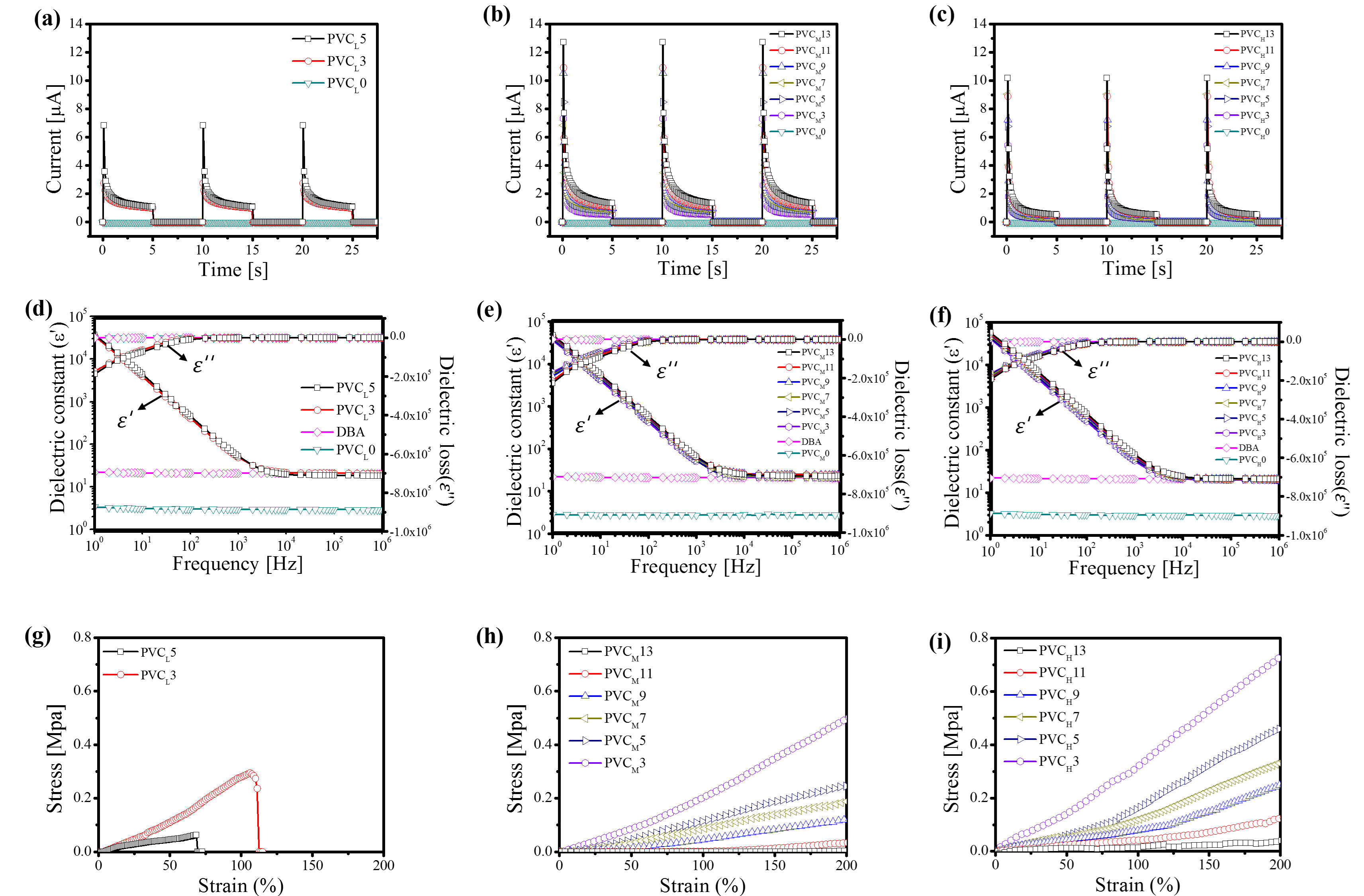


**Supplementary Figure S1: Electrical, dielectric, and mechanical properties of the plasticized PVC gels with different molecular weights of PVC polymer and amounts of DBA plasticizer.** A wide variety of physically crosslinked PVC gels were introduced by involving different weight ratios of PVC resin to DBA plasticizer. We evaluate electrical (a-c), dielectric (d-f), and mechanical (g-i) properties of the plasticized PVC gels prepared with different molecular weights of PVC polymer and amounts of DBA plasticizer to select optimal PVC gels as the electroactive PVC gel micro-lens.

**The lifetime (durability) of the PVC_H_11 gel micro-lens.**


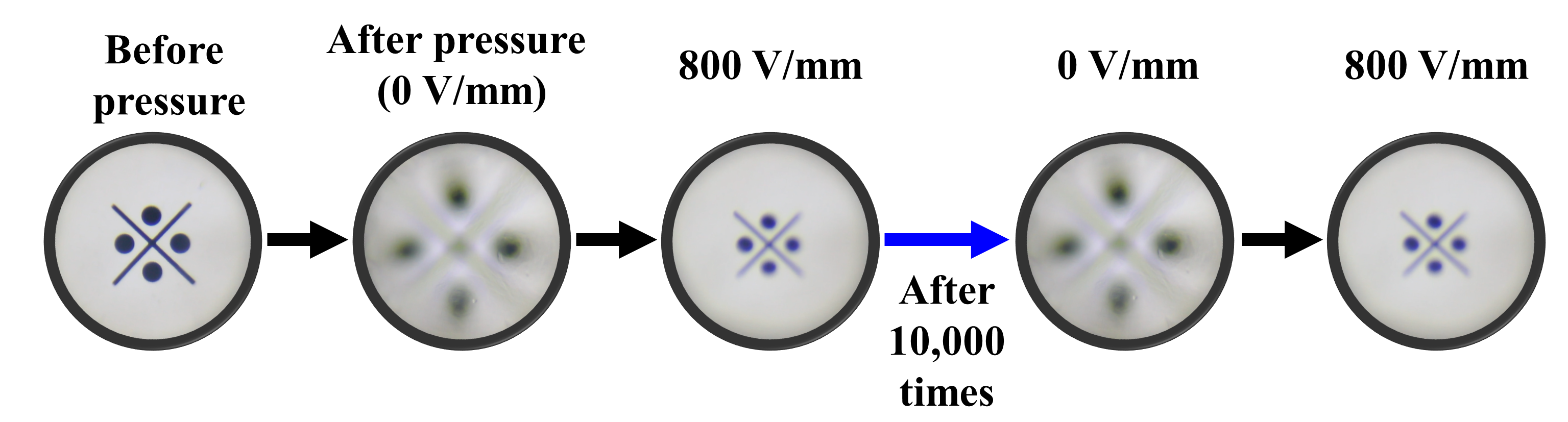


**Supplementary Figure S2: The target image observed from the microscope under alternative electric fields of 800 V/mm and 0 V/mm, after 10,000-actuation cycles of the PVC_H_11 gel micro-lens.** To verify the lifetime (durability) of the lens, alternative electric field of 800 V/mm and 0 V/mm were applied to the PVC_H_11 gel micro-lens. The electric field of 800 V/mm was applied to the proposed gel lens during 5s and then the electric field of 0 V/mm was provided to the lens for 5s. This procedure was conducted 10,000 times.

**SUPPLEMENTARY MOVIES**

**Supplementary Movie S1:** The structural design, operating principle, assembling procedure, and optical performance of bio-inspired PVC gel mciro-lenses were shown in Movie S1. The optical characteristics of micro-lenses based on the optimized non-ionic PVC gels (PVC_L_5, PVC_M_9, PVC_H_11) were systematically evaluated under an applied input voltage.
